# Unveiling the Influence of Activators on Stability and Pore Features of Foamed Concrete

**DOI:** 10.3390/ma18143320

**Published:** 2025-07-15

**Authors:** Yuanliang Xiong, Shiquan Wang, Liguo Ma, Tingcong Wang, Manling Zhou, Zhongshuai Hu, Zhenyu Wang

**Affiliations:** 1 School of Civil Engineering, Yantai University, Yantai 264005, China; 2 Lunan High Speed Railway Co., Ltd., Jinan 250000, China; 3 Qingdao Beiyang Architectural Design Co., Ltd., Qingdao 266100, China; 4 School of Energy and Constructional Engineering, Shandong Huayu University of Technology, Jinan 253034, China

**Keywords:** hybrid alkali-activated cement, foamed concrete, foam stability, pore structure

## Abstract

In this study, sodium hydroxide and calcium hydroxide are employed as activators to enhance the properties of foam concrete with hybrid alkali-activated cementitious material as the base mix. The effect of the activators on the properties of foam concrete is studied. The experimental results reveal that the presence of sodium hydroxide can also enhance the hydration rate of the base mix and increase the density of the pore wall in foamed concrete, thus enhancing the compressive strength of foamed concrete. Meanwhile, the addition of excessive sodium hydroxide may introduce too much water and increase the settlement of foamed concrete, thus resulting in a coarse and uneven pore structure. The settlement of the foam concrete with calcium hydroxide as an activator can be significantly increased, which is more related to the settlement of the matrix. The presence of calcium hydroxide could enhance dense pore walls, thus increasing the compressive strength and lowering water absorption.

## 1. Introduction

Foam concrete has rapidly gained market recognition as a building material due to its advantageous properties, including lightweight characteristics, high strength, superior thermal insulation, and cost-effectiveness [1,2,3,4]. However, the traditional foam concrete uses Portland cement as the main material, which is expensive and does not conform to the concept of green environmental protection, limiting its application [5,6,7]. However, alkali-activated foam concrete mostly uses low-carbon solid wastes such as slag and fly ash as the main raw materials, which can make full use of solid waste resources and has the advantages of low cost and green environmental protection [8].

In alkali-activated foam concrete formulations, certain industrial waste slags often exhibit elemental deficiencies. Even with optimized alkali activator dosage, these slags may still fail to meet the stoichiometric ratios required for geopolymer formation. Consequently, slag is commonly combined with supplementary solid aluminosilicate sources [9,10,11]. The hydration of Portland cement supplies critical calcium hydroxide for slag reactivity, whereas slag concurrently accelerates cement hydration via dilution and nucleation mechanisms [12,13,14]. The foam concrete synthesized by integrating ordinary Portland cement with slag may present distinctive performance. In hybrid alkali-activated foam concrete, selecting an appropriate activator and its dosage is one of the effective methods to improve its stability and optimize its pore structure [15,16,17,18,19,20]. However, the performance of hybrid alkali-activated foam concrete with cement, slag, and different activators needs to be further studied.

Generally speaking, foam concrete has two pore structures. One is a structural pore, including gel pore (less than 5 nm), capillary pore (more than 200 nm), and transition pore. The other is a bubble pore formed after foam is added. The bubble pore is often divided into closed round pores, semi-closed pores, and connected pores due to production process, materials, and other reasons [21,22]. Because the structural pore is much smaller than the bubble pore in size and independent, the bubble pore has a relatively large impact on the compressive strength and water absorption of foam concrete. In the alkali excitation environment, the direct effect of the activator on foam and the nature of the matrix are the key factors affecting the stability of foam concrete and its pore structure characteristics. Early research shows that, with the increase in alkali content, foam will cause structural instability and damage due to coagulation and coarsening, but the addition of an appropriate amount of alkaline activator will improve the viscosity of the matrix and enhance the binding force on foam, thereby inhibiting the instability of foam and forming a better structured bubble hole [23,24]. Therefore, the pore structure of foam concrete can be optimized by adding an appropriate alkaline activator in the matrix. However, many studies have explained the positive correlation between the fineness of pore structure and the performance of foam concrete [22,25], and whether the pore wall compactness is more important than the fineness of pore structure needs further exploration.

In this study, the effects of sodium hydroxide and calcium hydroxide on the early stability, pore structure, compressive strength, and water absorption of foam concrete were studied. The mechanism of its action was studied using laser displacement sensors, SEM, and XRD.

## 2. Experiments

### 2.1. Materials

P O. 42.5 ordinary Portland cement and S95-grade mineral powder are used as matrix materials, with a specific surface area of 400 m^2^/kg for S95-grade mineral powder. The chemical composition of P O. 42.5 ordinary Portland cement and slag is shown in Table 1. The nano-modified synthetic foaming agent is used for physical foaming. The calcium hydroxide analytical reagent and sodium hydroxide analytical reagent (purity ≥ 99%) (Yantai Luyang Chemical with Chinese medicine, Yantai, China) are used as activators.

### 2.2. Sample Preparation

The mix proportion and density grade of foam concrete are shown in Table 2. Firstly, dilute the foaming agent with deionized water in a mass ratio of 1:300 and let it stand for 24 h before use. Dilute sodium hydroxide with deionized water in a mass ratio of 1:2 and let it stand for 24 h before use. The FC-Ref group involves dry mixing the cementitious material at a speed of 60 r/min for 2 min, followed by adding water and stirring for 4 min. Use LC-01B to prepare foam, then introduce foam into the slurry, and mix for 2–3 min at the speed of 60–120 r/min to prepare foam concrete. For the group mixed with sodium hydroxide, first, dry mix the cementitious material at a ratio of 60 r/min for 2 min, then add water (water in the sodium hydroxide solution has been removed) and stir for 4 min. Within the first 1 min after adding water and stirring, add the sodium hydroxide solution. The introduction process of foam is the same as above. For the group mixed with calcium hydroxide, dry mix the powdered calcium hydroxide and cementitious material at the rate of 60 r/min for 2 min, and then add water to mix for 4 min. The introduction process of foam is the same as above. The mix proportions of foamed concrete are listed in Table 2. The 3% NaOH, 5% NaOH, 7% NaOH, and 7% Ca (OH)_2_ were used as the activators based on references [23,26].

### 2.3. Testing Methods

#### 2.3.1. Yield Stress of Matrix

**Figure 1:**
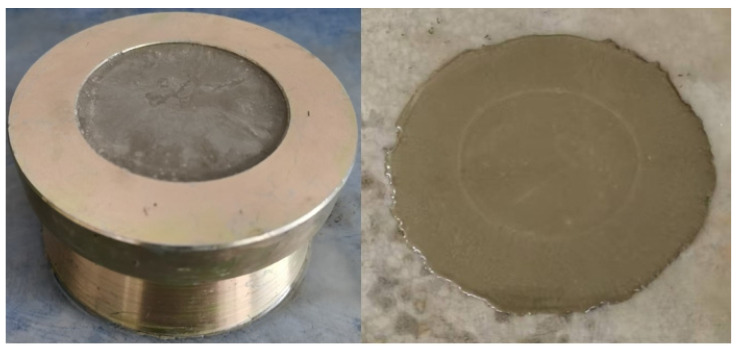
Truncated circular mold.

According to the method described in references [22,24,25], the yield stress was experimentally tested using a truncated circular mold (with a bottom diameter of 100 mm), as shown in Figure 1, where the freshly mixed matrix was poured into the truncated circular mold at different times until it was filled. Then, vertically lift the truncated cone circular mold and wait for the slurry to flow for 2 min before testing the unfolded diameter of the slurry at different times. Then, use formula (a) for calculation, which depends on the pouring volume (*V*) and density (*ρ*). Calculate the yield stress using the acceleration of gravity (*g*) and the expansion radius (*R*). The yield stress at different times for each group is recorded and calculated.

(1) $$\tau =\frac{225\rho gV^{2}}{128\pi^{2}R^{5}}$$

#### 2.3.2. The Settlements

**Figure 2:**
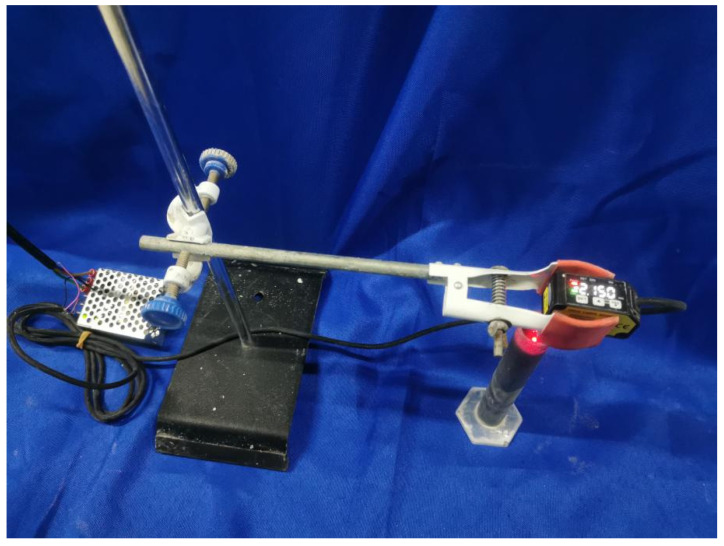
The experimental configuration for quantifying the sedimentation.

The settlements recorded by the laser distance sensor of JINGJIAKE BX-LV30 (Anyi Optoelectronics, Technology Co., Ltd., Suzhou, China) were used to investigate the stability of the base mix and foamed concrete. Pour the freshly mixed sample into a cylinder and measure the settlement of the sample, as shown in Figure 2. The settlement was measured at different times, and the accuracy of the measured settlement distance was 0.01 mm.

#### 2.3.3. Setting Time

The initial setting time of the foam concrete matrix was tested by the Vicat apparatus base on the reference [23]. The cement paste was put into the test mold placed on the glass substrate, then the mold was placed in a moisture curing box for curing at 20 ± 1 °C, with relative humidity (RH) > 90%.

#### 2.3.4. XRD Detection

An X-ray diffractometer (Bruker D8 Advance, Bruker Corporation, Berlin, Germany) was used to determine the hydration products of the sample. Before the experiment, all samples were first crushed and passed through a 200-mesh sieve for homogenization. Then, the powder was dried at 60 °C to a constant weight. The scanning scope was adjusted to 5–70°, accompanied by a scanning rate of 0.15 s per step.

#### 2.3.5. SEM-EDS Testing

The SEM (FEI 3D, FEI Corporation, Hillsboro, OR, USA) was used to record the microstructure of foamed concrete. To enhance conductivity, a gold layer roughly 10–50 nm thick was applied to the samples prior to the test. Then, the samples were scanned 100 times and 200 times by a high-power electron microscope.

#### 2.3.6. Bubble Evolution and Pore Structure

The bubble evolution process of fresh foam coagulation is recorded by an optical microscope (RY605, Shanghai Renyue Electronic Technology Co., Ltd., Shanghai, China). When exploring the hole structure of foam concrete, saw off the middle 30 mm part of the PVC pipe with foam concrete (30 mm in diameter and 300 mm in length), polish the sawn section with 1200 mesh sandpaper, and use adhesive tape to remove the ash falling from the section.

#### 2.3.7. Hardened Foamed Concrete Properties

In order to test the compressive strength of foam concrete, the fresh foam concrete is poured into a mold with an internal size of 70.7 mm × 70.7 mm × 7.07 mm, and then demolded 24 h later. The demolded cube test block is placed in the standard curing room for curing. Before testing, dry the sample in a 60 °C oven to a constant weight, then let it stand at room temperature. Then, use a pressure testing machine to load to test its compressive strength. The measurement of water absorption is performed by drying the sample cured for 28 days at 60 °C, weighing the dry weight before the test, and then putting it into water to test its mass at different times, so as to calculate the water absorption. Three samples were used for each group of compressive strength and water absorption experiments.

## 3. Results and Discussion

### 3.1. Effect of Activator on Matrix Stability

**Figure 3:**
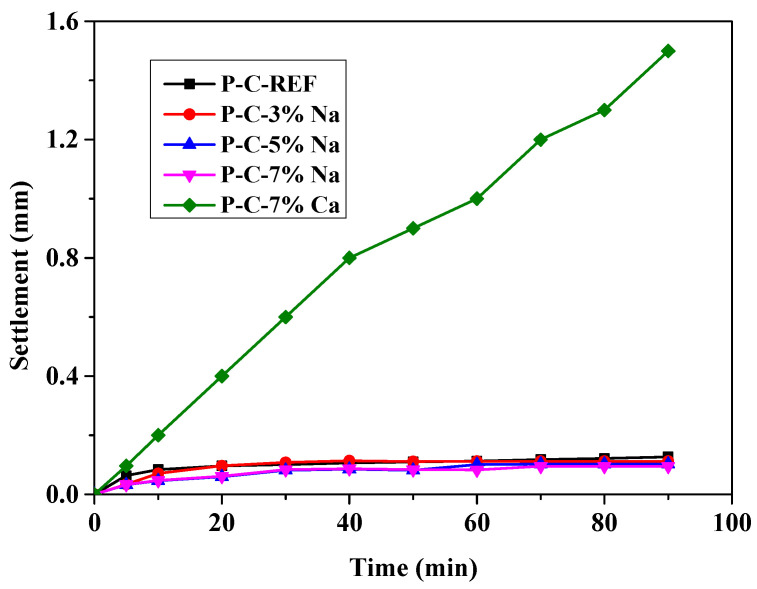
Settlement of base mixes.

Figure 3 shows the influence of the activators on matrix settlement. The matrix settlement of the three groups using sodium hydroxide as the activator tends to be similar and smaller. The matrix settlement of the FC-Ref group is similar to the group containing sodium hydroxide, while the matrix settlement of the group containing calcium hydroxide is much higher than that of the other groups.

### 3.2. Effect of Activator on Stability of Foam Concrete

**Figure 4:**
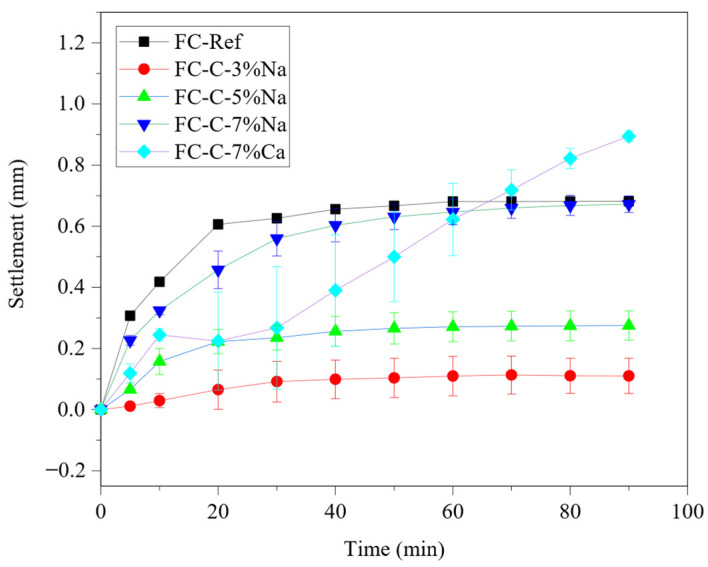
Settlement of fresh foamed concretes.

Figure 4 shows the influence of the activators on the settlement in foam concrete. The final settling amounts of FC-Ref, FC-C-3% Na, FC-C-5% Na, FC-C-7% Na, and FC-C-7% Ca are 0.682 mm, 0.110 mm, 0.275 mm, 0.672 mm, and 0.895 mm, respectively. In the group mixed with sodium hydroxide, with the increase of the amount of activator, the settlement of foam concrete enhances. The development of yield stress for different groups of matrices at different times is shown in Table 3. The FC-C-3% Na possesses the highest yield stress compared with FC-Ref, FC-C-3% Na, FC-C-5% Na, FC-C-7% Na. It can be seen that the yield stress of the base mix may be the main reason affecting the stability of foamed concrete.

**Figure 5:**
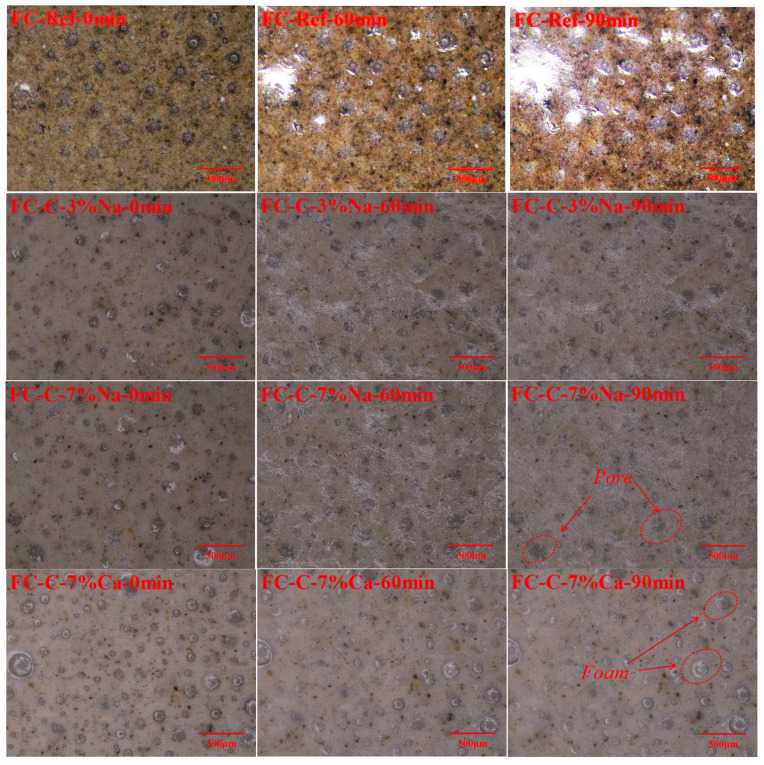
Bubble evolution process of fresh hybrid alkali-activated foamed concrete.

The introduction of sodium hydroxide could decrease the fluidity of cement pastes, thus increasing the yield stress [27], while the addition of excessive sodium hydroxide may introduce too much water, thus increasing the settlement of foam concrete. However, the stability of the calcium hydroxide group is negatively related to the yield stress of the base mix, which may be due to the larger settlement of the matrix during the first 90 min of settlement. According to Figure 5, it can be seen that, except for the FC-C-7% Ca group, almost all bubbles in the other groups evolved into pores in the first 90 min. However, there were still many unbroken surface bubbles in the FC-C-7% Ca group within the first 90 min. It can be inferred that the settlement of foam concrete of FC-C-7% Ca group is greatly affected by the settlement of the matrix in the first 90 min.

### 3.3. Foam Concrete Pore Structure

**Figure 6:**
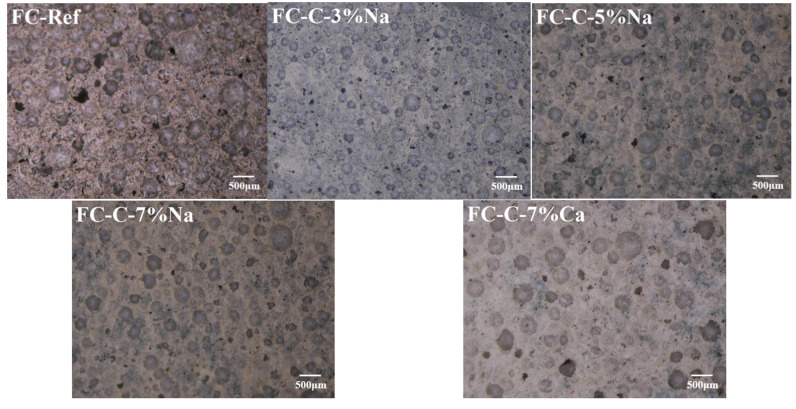
2D images of HAAFC.

**Figure 7:**
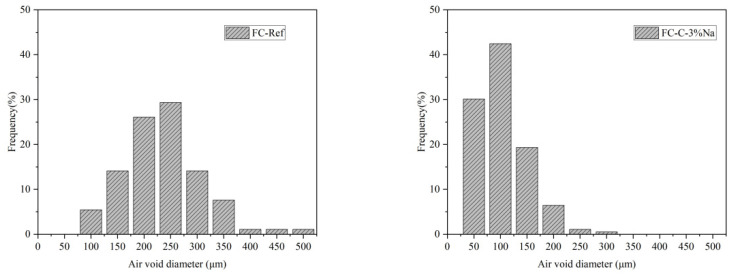

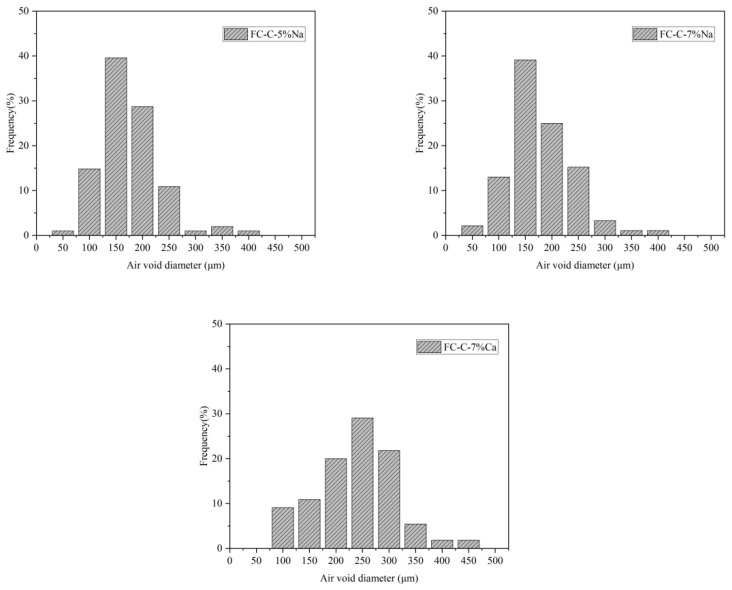
Pore size distribution of foamed concrete.

Figure 6 shows the picture of the foam concrete pore structure of each group. Image Pro Plus software (IPWIN60) was used to perform aperture size statistics. Figure 7 illustrates the distribution of pore sizes in each cohort of aerated concrete. It can be seen from Figure 6 that the foam concrete pore size of the control group and FC-C-7Ca group is larger. In the group mixed with sodium hydroxide, the pore structure size of the FC-C-3% Na group is relatively fine. With the increase of sodium hydroxide content, the pore size of foam concrete gradually increases. The proportion of pore size of the FC-Ref ranges from 150 to 250 μm is 55.44%; the corresponding proportion of FC-C-3% Na, FC-C-5% Na, and FC-C-7% Na is 72.59%, 68.31%, and 64.13%. The proportion of pore size of the FC-C-7% Ca ranges from 200 to 300 μm is 50.91%. Evidently, as the concentration of sodium hydroxide escalates, the pore diameter of foam concrete expands, accentuating the heterogeneity in their sizes. The pore diameter of FC-C-7% Ca is larger and more uneven than that of FC-C-7% Na. This corresponds to the settlement of foam concrete measured earlier, that is, the larger the settlement amount of fresh foam concrete, the larger the pore structure size formed, and the more uneven. However, according to the previous foam concrete settlement and matrix settlement experiments, the settlement of FC-C-7Ca foam concrete was greatly affected by the matrix settlement in the first 90 min. According to the evolution process of bubbles in foam concrete, the bubbles in this group of foam concrete did not break in a larger scale than other groups in the first 90 min, which is obviously contradictory to the pore structure with larger pore size in this group, so it needs to be explored from other aspects. Compared with other groups, by observing the settlement diagram of foam concrete in this group, the settlement process of fresh foam concrete in this group did not end in the first 90 min, so it is more likely that bubbles will continue to break and coalesce on a large scale in the later period. The duration required for the matrix to set significantly impacts the stability of foam concrete [28]. Therefore, the matrix setting time of each group was tested, and the matrix setting times of FC-Ref, FC-C-3% Na, FC-C-5% Na, FC-C-7% Na, and FC-C-7% Ca groups were 422 min, 281 min, 230 min, 143 min, and 545 min, respectively. It can be seen that the matrix of the FC-C-7% Ca group has the longest setting time, which means there is more time for the bubbles to continue to coalesce and rupture. This may be the reason why the aperture size of this group is larger and the uniformity is smaller [23].

### 3.4. Pore Wall of Foam Concrete

**Figure 8:**
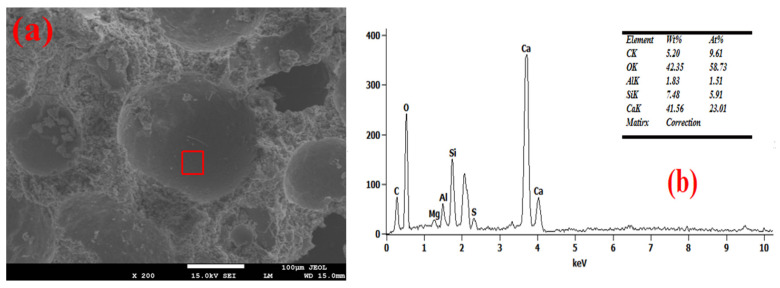
Microstructure of FC-Ref. (**a**) SEM and (**b**) EDS spectrum.

**Figure 9:**
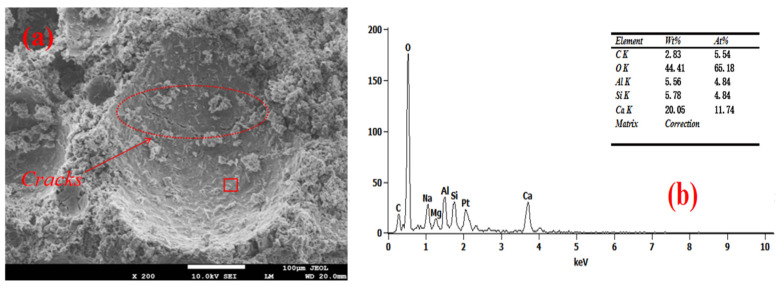
Microstructure of FC-C-3%Na. (**a**) SEM and (**b**) EDS spectrum.

**Figure 10:**
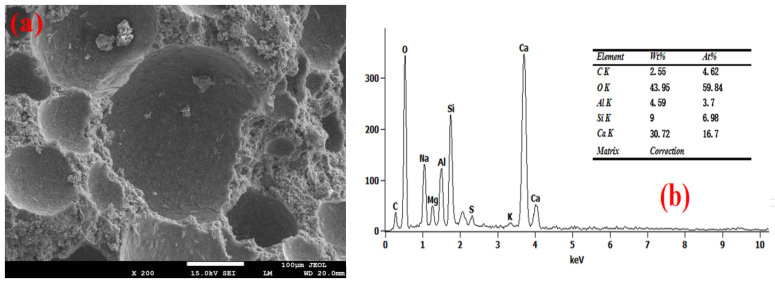
Microstructure of FC-C-5%Na. (**a**) SEM and (**b**) EDS spectrum.

**Figure 11:**
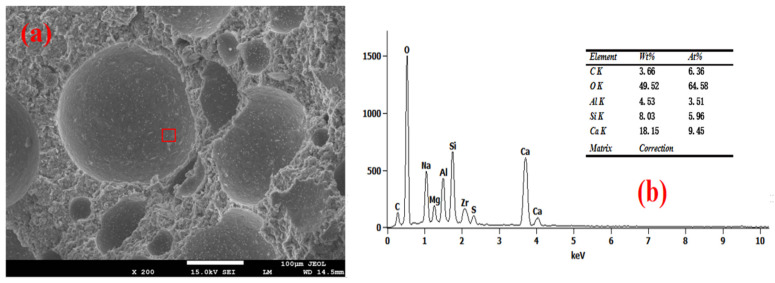
Microstructure of FC-C-7%Na. (**a**) SEM and (**b**) EDS spectrum.

**Figure 12:**
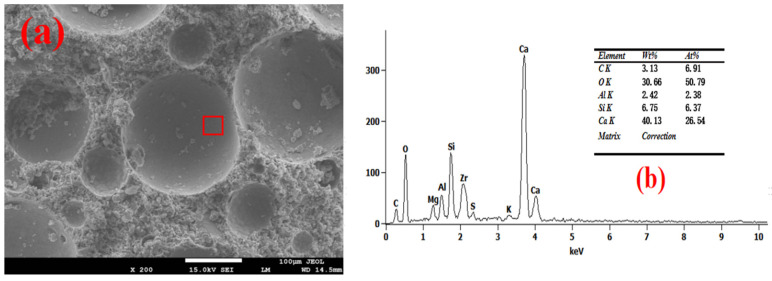
Microstructure of FC-C-7%Ca. (**a**) SEM and (**b**) EDS spectrum.

The SEM-EDS test results of the foam concrete hole wall are shown in Figure 8, Figure 9, Figure 10, Figure 11 and Figure 12. The red square of SEM pictures were used to test the EDS spectrum. From the SEM-EDS test results, it can be seen that among the three groups doped with sodium hydroxide, the pore walls of the FC-C-3% Na group have obvious cracks, and there are a large number of capillary pores in the matrix around the pore walls. With the increase of sodium hydroxide content, the pore wall of foam concrete is significantly more compact, and the pores around the pore wall are significantly reduced. This is because lower hydroxyl content will lead to less leached silicon monomer in the slag, which will slow the dissolution rate of the matrix material and produce more capillary channels and less hydration products with N-A-S-H gel structure [29,30,31]; consequently, the hole wall cannot be effectively fille, resulting in a thinner hole wall, so the hole wall is prone to cracks and holes.

With the increase of sodium hydroxide content, more products with N-A-S-H gel structure fill the pore wall, which makes the pore wall and matrix more dense. From the graph, it can be seen that compared with FC-C-7% Na, the pore walls of the FC-Ref and FC-C-7Ca groups are smoother and smoother, with higher density. For FC-C-7Ca, this is because more hydration products with the C-A-S-H structure will be produced under the alkaline action of calcium hydroxide [12]. According to molecular dynamics simulation, the C-a-S-H gel system is more dense than the N-A-S-H gel system [32], and calcium hydroxide reacts with nano alumina in the foam to generate calcium aluminate to fill the foam gas–liquid interface [33], which may be the reason why the pore wall of FC-C-7Ca group is smoother and smoother. In the FC Ref group, only ordinary Portland cement is used as the base material. Ordinary Portland cement has higher active ingredients than mineral powder. Among them, C_3_S, C_2_S, C_3_A, and C_4_AF can fully hydrate with water to produce more C_3_H_2_S_3_ and C_3_AH_6_ [12], so as to fill the internal hole wall of foam concrete, making the hole wall more smooth and flat.

### 3.5. Hydration Process of Foamed Concrete

**Figure 13:**
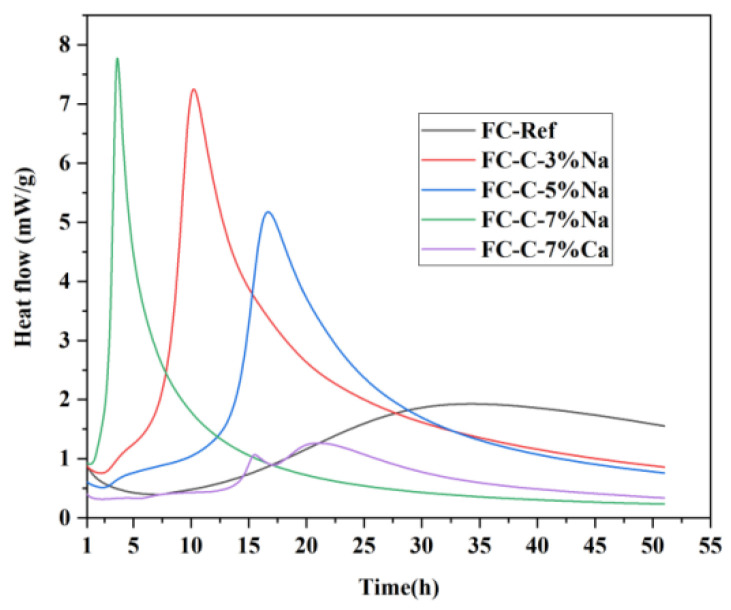
Cumulative heat release of foamed concrete.

Figure 13 describes the hydration process of foamed concrete. In FC-Ref, the hydration rate of slag is initially extremely slow. With the addition of cement, the formation of calcium–silicate–hydrate (C-S-H) and calcium hydroxide (Ca(OH)_2_) can be accelerated, thereby promoting the hydration process of slag [34]. An appropriate amount of NaOH can further speed up the hydration of C_3_S and C_2_S, while also raising the hydration degree. Additionally, it can bring forward the hydration exothermic peak of C_3_S [35].

### 3.6. Compressive Strength and Water Absorption Rate

**Figure 14:**
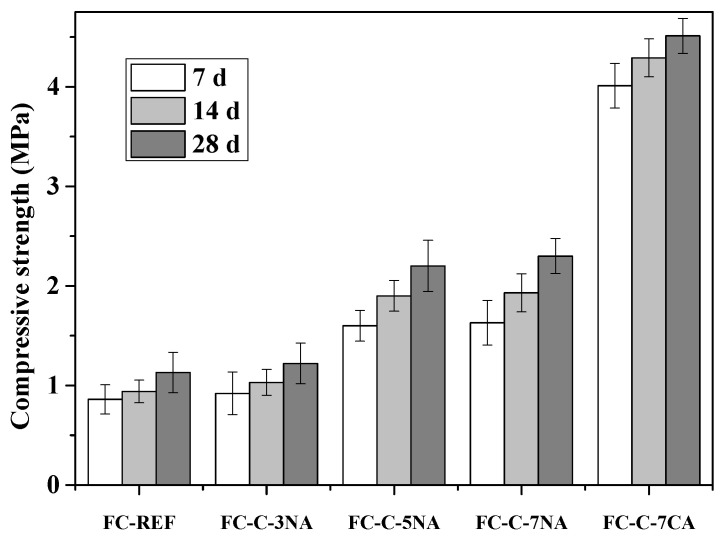
Compressive strength of foamed concrete.

Figure 14 elucidates the impact of the activator on the compressive strength of foam concrete. The 28 d compressive strength of FC Ref, FC-C-3% Na, FC-C-5% Na, FC-C-7% Na, and FC-C-7% Ca are 1.08 MPa, 1.15 MPa, 2.18 MPa, 2.31 MPa, and 4.78 MPa, respectively. The seven-day compressive strength of the FC-C-7% Ca group is 3.96 MPa. Evidently, the FC-C-7% Ca group exhibits a superior initial compressive strength compared to the FC-Ref group.

**Figure 15:**
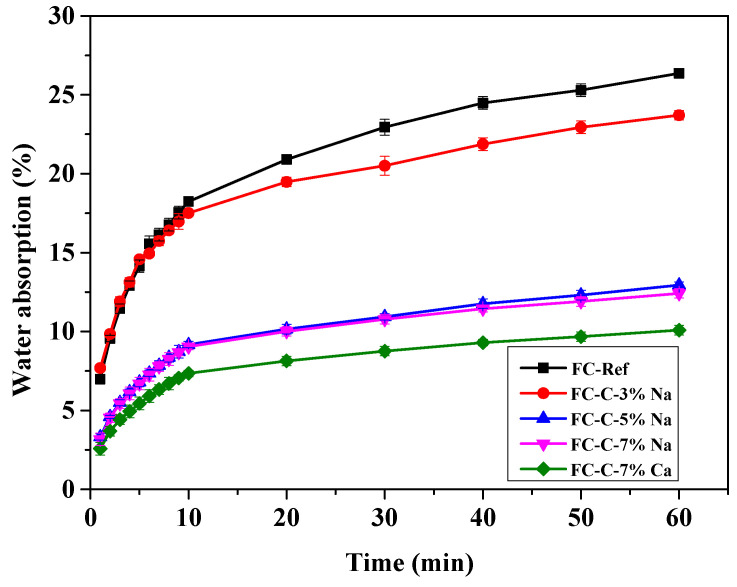
Water absorption of foamed concrete.

The water absorption of the activator to foam concrete is shown in Figure 15. The water absorption rates after 60 min for FC Ref, FC-C-3% Na, FC-C-5% Na, FC-C-7% Na, and FC-C-7% Ca are recorded as 25.12%, 24.40%, 13.46%, 12.76%, and 9.06%, respectively. It can be seen that under the alkali excitation environment, with the increase of sodium hydroxide content, the water absorption of foam concrete gradually decreases. Compared with the three groups with sodium hydroxide, the water absorption of foam concrete with calcium hydroxide is lower. The foam concrete with 7% calcium hydroxide has relatively dense pore walls, which may be the reason for higher compressive strength and lower water absorption [36].

## 4. Conclusions

In this study, different types of activators and their admixtures are used to improve the performance of foam concrete. The mechanism of action was studied using displacement sensors, optical microscopes, and SEM-EDS. The study yields several key findings:

1. The presence of sodium hydroxide can also enhance the hydration rate of the base mix and increase the density of the pore wall in foamed concrete, thus enhancing the compressive strength of foamed concrete.
2. The addition of excessive sodium hydroxide may introduce too much water and increase the settlement of foamed concrete, thus resulting in a coarse and uneven pore structure.
3. The presence of 7% calcium hydroxide could enhance dense pore walls, thus increasing the compressive strength and reducing the water absorption.

## Figures and Tables

**Table 1:** Compositions of cement, wt%.

	CaO	SiO_2_	Al_2_O_3_	Fe_2_O_3_	Na_2_O	MgO	SO_3_	LOI	K_2_O
P O. 42.5	51.42	24.99	8.26	4.03	0.11	3.71	2.51	4.32	0.65
GBFS	35.58	35.10	16.32	0.69	9.32	1.17	0.41	0.49	0.92

**Table 2:** Mix designs of foamed concrete.

Mix	Target Density (kg/m^3^)	OP (kg)	GBFS (kg)	Water (kg)	NaOH (kg)	Ca(OH)_2_ (kg)	Actual Foam (m^3^)
FC-Ref	800	-	498.13	249.07	14.94	-	0.57
FC-C-3Na	800	99.63	398.50	249.07	14.94	-	0.57
FC-C-5Na	800	99.63	398.50	249.07	24.91	-	0.57
FC-C-7Na	800	99.63	398.50	249.07	34.87	-	0.56
FC-C-7Ca	800	99.63	398.50	249.07	-	34.87	0.56

**Table 3:** The dispersion and yield stress of cement pastes at various time intervals.

Time (min)	Spread (mm)					Yield Stress (Pa)				
	P-Ref	P-C-3Na	P-C-5%Na	P-C-7%Na	P-C-7%Ca	P-Ref	P-C-3%Na	P-C-5%Na	P-C-7%Na	P-C-7%Ca
0	245	178	191	209	151	13.66	65.58	46.20	29.51	>100
10	215	167	184	192	143	26.25	90.22	55.68	45.10	>100
30	198	150	166	175	134	39.62	>100	93.17	71.70	>100
60	192	126	152	155	120	46.20	>100	>100	>100	>100

## Data Availability

The original contributions presented in the study are included in the article, further inquiries can be directed to the corresponding authors.
